# Ceramic Heads Decrease Metal Release Caused by Head-taper Fretting and Corrosion

**DOI:** 10.1007/s11999-015-4683-1

**Published:** 2016-02-04

**Authors:** Sevi B. Kocagoz, Richard J. Underwood, Daniel W. MacDonald, Jeremy L. Gilbert, Steven M. Kurtz

**Affiliations:** School of Biomedical Engineering, Science, and Health Systems, Drexel University, Philadelphia, PA USA; Exponent Inc, Philadelphia, PA USA; Syracuse Biomaterials Institute and Department of Biomedical and Chemical Engineering, Syracuse University, Syracuse, NY USA; Implant Research Center, School of Biomedical Engineering, Science, and Health Systems, Drexel University, 3401 Market Street, Suite 345, Philadelphia, PA 19104 USA

## Abstract

**Background:**

Metal release resulting from taper fretting and corrosion is a clinical concern, because wear and corrosion products may stimulate adverse local tissue reactions. Unimodular hip arthroplasties have a conical taper between the femoral head (head bore taper) and the femoral stem (stem cone taper). The use of ceramic heads has been suggested as a way of reducing the generation of wear and corrosion products from the head bore/stem cone taper junction. A previous semiquantitative study found that ceramic heads had less visual evidence of fretting-corrosion damage compared with CoCr heads; but, to our knowledge, no studies have quantified the volumetric material loss from the head bore and stem cone tapers of a matched cohort of ceramic and metal heads.

**Questions/purposes:**

We asked: (1) Do ceramic heads result in less volume of material loss at the head-stem junction compared with CoCr heads; (2) do stem cone tapers have less volumetric material loss compared with CoCr head bore tapers; (3) do visual fretting-corrosion scores correlate with volumetric material loss; and (4) are device, patient, or intraoperative factors associated with volumetric material loss?

**Methods:**

A quantitative method was developed to estimate volumetric material loss from the head and stem taper in previously matched cohorts of 50 ceramic and 50 CoCr head-stem pairs retrieved during revision surgery for causes not related to adverse reactions to metal particles. The cohorts were matched according to (1) implantation time, (2) stem flexural rigidity, and (3) lateral offset. Fretting corrosion was assessed visually using a previously published four-point, semiquantitative scoring system. The volumetric loss was measured using a precision roundness machine. Using 24 equally spaced axial traces, the volumetric loss was estimated using a linear least squares fit to interpolate the as-manufactured surfaces. The results of this analysis were considered in the context of device (taper angle clearance, head size, head offset, lateral offset, stem material, and stem surface finish) and patient factors that were obtained from the patients’ operative records (implantation time, age at insertion, activity level, and BMI).

**Results:**

The cumulative volumetric material losses estimated for the ceramic cohort had a median of 0.0 mm^3^ per year (range, 0.0–0.4 mm^3^). The cumulative volumetric material losses estimated for the CoCr cohort had a median of 0.1 mm^3^ per year (range, 0.0–8.8 mm^3^). An order of magnitude reduction in volumetric material loss was found when a ceramic head was used instead of a CoCr head (p < 0.0001). In the CoCr cohort, the femoral head bore tapers had a median material loss of 0.02 mm^3^ (range, 0.0–8.7 mm^3^) and the stem cone tapers had a median material loss of 0.0 mm^3^ (range, 0.0–0.32 mm^3^/year). There was greater material loss from femoral head bore tapers compared with stem cone tapers in the CoCr cohort (p < 0.001). There was a positive correlation between visual scoring and volumetric material loss (Spearman’s ρ = 0.67, p < 0.01). Although visual scoring was effective for preliminary screening to separate tapers with no or mild damage from tapers with moderate to severe damage, it was not capable of discriminating in the large range of material loss observed at the taper surfaces with moderate to severe fretting-corrosion damage, indicated with a score of 3 or 4. We observed no correlations between volumetric material loss and device and patient factors.

**Conclusions:**

The majority of estimated material loss from the head bore-stem cone junctions resulting from taper fretting and corrosion was from the CoCr head bore tapers as opposed to the stem cone tapers. Additionally, the total material loss from the ceramic cohort showed a reduction in the amount of metal released by an order of magnitude compared with the CoCr cohort.

**Clinical Relevance:**

We found that ceramic femoral heads may be an effective means by which to reduce metal release caused by taper fretting and corrosion at the head bore-stem cone modular interface in THAs.

**Electronic supplementary material:**

The online version of this article (doi:10.1007/s11999-015-4683-1) contains supplementary material, which is available to authorized users.

## Introduction

Fretting corrosion at the head-stem modular junction has reemerged as a clinical concern for large head metal-on-metal (MoM) and metal-on-polyethylene (MoP) THAs [6, 7, 21]. Some studies have suggested that wear and corrosion products may be a factor in stimulating adverse local tissue reactions [5, 10, 21]. Most modern designs of THA implants use a modular junction where the surfaces of the femoral head bore taper and the femoral stem cone taper interlock. Visual evidence of taper corrosion at the modular junctions has been observed in 44% to 96% of components in studies investigating large-head MoM and MoP bearings [9, 11, 14, 21]. There is interest in investigating the device and patient factors that may be associated with fretting and corrosion of modular tapers [11, 17, 18] to establish better treatment options [6].

Ceramic femoral heads have been proposed as a way to mitigate taper corrosion [12, 17]. We previously studied a matched cohort of 50 ceramic and 50 metal head-stem pairs using semiquantitative fretting and corrosion damage scores [17]. Visual damage scoring has been established as a useful method to rank the severity of fretting corrosion in an available group of retrievals [11]. However, this method is not always sufficient to assess fretting-corrosion damage, particularly in components that have severe corrosion, and the amount of material loss varies widely [15]. Therefore, it may be necessary in some cases to quantify material loss by direct measurements. We developed and validated quantitative methods to estimate taper angle clearance from retrieved head bore and stem cone tapers [16]. The material loss resulting from taper corrosion has been estimated in large-head MoM hip bearings [18, 19]; however, to our knowledge, no studies have been published that estimate the volume of material loss from ceramic-on-polyethylene (CoP), ceramic-on-ceramic (CoC), or MoP bearings. Furthermore, the relationship between material loss and tolerances of the head-stem modular connection are poorly understood.

We sought to address the following research questions: (1) Do ceramic heads result in less volume of material loss at the head-stem junction compared with CoCr heads; (2) do stem cone tapers have less volumetric material loss compared with CoCr head bore tapers; (3) do visual fretting-corrosion scores correlate with volumetric material loss; and (4) are device, patient, or intraoperative factors associated with volumetric material loss?

## Materials and Methods

### Study Design and Clinical Information

We previously matched cohorts of 50 retrieved ceramic head-stem pairs with 50 CoCr head-stem pairs that were used in earlier studies to investigate whether there was a correlation between visual taper corrosion and head material [17] and taper angle clearance [16]. The most prevalent reasons for revision in this study were loosening, infection, fracture, and pain (Table 1), and not for reasons relating to corrosion or metal debris. Composite fretting-corrosion damage for the cohorts in this study was characterized using a previously published 4-point scoring method [14], which was modified from the original method of Goldberg et al. [11]. Scoring of the cohorts used in this study was done by three independent observers (SBK, DWM, JAH) who characterized the damage on the tapers from a scale of 1 to 4 with 1 being the least severe and 4 being the most severe [11]. A sample size of 100 was based on a power calculation that allowed our study to have 99% power to detect a difference of 1 in fretting-corrosion scores between the metal and ceramic cohorts [17]. Design, patient, and revision information was available for all retrievals through a review of the operative notes that were provided by the surgical center where the revision was performed (Table 1).

**Table 1:** Patient and device information for ceramic and CoCr cohorts*

Variable	Ceramic cohort	CoCr cohort	p value (Mann-Whitney U)
Patient Information (mean ± SD)			
Implantation time (years)	3 ± 3	3 ± 2	0.7
Age at implantation (years)	52 ± 10	57 ± 14	0.03
Gender (F:M) (number (%))	17 (34%)	25 (50%)	0.11
BMI (kg/m^2^)	30 ± 7	30 ± 7	0.91
UCLA Activity Score	6 ± 2	5 ± 2	0.65
Reason for revision (number of components)			0.065 (Pearson)
Loosening	28	22	
Infection	13	20	
Fracture	1	4	
Pain	2	1	
Other	6	3	
Stem design (number of components)			0.34 (Pearson)
Accolade^TM^ (Stryker^®^, Mahwah, NJ, USA)	28	27	
Zimmer^®^ M/L Taper (Zimmer, Warsaw, IN, USA)	3	4	
VerSys^®^ (Zimmer, Warsaw, IN, USA)	2	4	
Tri-Lock^®^ (Depuy Synthes, West Chester, PA, USA)	2	2	
Corail^®^ (Depuy Synthes, West Chester, PA, USA)	3	3	
Other	12	10	

* Previously matched cohorts [17].

As described previously [17], the cohorts were matched according to: (1) implantation time; (2) stem flexural rigidity; and (3) lateral offset. The flexural rigidity of each stem is calculated by multiplying the elastic modulus (E) of the stem material and second moment of area (I). The moment of area $I = \frac{\pi }{4}r^{2}$ was determined using the radius of the stem cone taper (r) at the distal end where the trunnion exits the bore. The stem materials for the ceramic cohort are: CoCr alloy (n = 6, E = 220 GPa); Ti-6Al-4V alloy (n = 16, E = 110 GPa); and TMZF^®^ alloy (Stryker, Mahwah, NJ, USA) (n =28, E = 79.5 GPa). The stem materials for the CoCr cohort are: CoCr alloy (n = 8, E = 220 GPa); Ti-6Al-4V alloy (n = 17, E = 110 GPa); and TMZF^®^ alloy (n = 25, E = 79.5 GPa). The ceramic and CoCr cohorts had similar head diameters (median = 32 mm, mean = 33 mm for both cohorts, p = 0.65, Mann-Whitney U; ceramic cohort range, 28–36 mm; CoCr cohort range, 22–40 mm). On average, the patients in the ceramic cohort were 5 years younger than those in the CoCr cohort. The ceramic cohort included CoP (n = 41) and CoC bearings (n = 9), while the CoCr cohort included only MoP (n = 50) bearings. This study did not include components with large-head MoM bearings or modular femoral stems or necks. The reasons for revision included loosening (ceramic cohort, n = 28; CoCr cohort, n = 22), infection (ceramic cohort, n = 13; CoCr cohort, n = 20), periprosthetic fracture (ceramic cohort, n = 1; CoCr cohort, n = 3; component fracture CoCr cohort, n =1), pain (ceramic cohort, n = 2; CoCr cohort, n = 1), and other (ceramic cohort, n = 6; CoCr cohort, n = 3). No components were reported to have a revision reason involving pseudotumor formation or metallosis (Table 1).

### Estimation of Material Loss From Head Bore Tapers

For this study, we used a previously developed quantitative method to estimate material loss from femoral head tapers. The taper surface was measured using a roundness machine (Talyrond^®^ 585, Taylor Hobson Ltd, Leicester, UK) equipped with a diamond stylus. The axis of the taper was aligned with the axis of rotation of the Talyrond^®^ rotation using the automatic centering and leveling routine. A total of 24 equally spaced axial profiles were measured on the surface of each head taper.

The profiles were analyzed and the volume of material loss was estimated using a customized MATLAB^®^ (MathWorks^®^ Inc, Natick, MA, USA) script. The volumetric material loss was estimated from the following steps: (1) the user identified regions of “as-manufactured” surface on each profile; (2) a least-squares line was fitted through as-manufactured regions to establish the presumed as-manufactured surface profile in the areas of material loss; (3) integrated areas of material loss were calculated using the spacing between each measured data point and the distance between the measured surface and the estimated as-manufactured surface; (4) area of material loss was used to calculate the volume of a partial annulus based on the taper local radius and spacing to the next axial profile; and (5) all partial annuli were summed to estimate the volume of material loss in the taper (Appendix 1. Supplemental material is available with the online version of *CORR*^®^).

During method development for estimation of volumetric material loss we compared our method with gravimetric measurements of material loss for a cohort of never-implanted (exemplar) femoral heads and taper adapter sleeves with artificial material loss. The volume and pattern of artificial material loss in the exemplars was representative of material loss observed in retrieved femoral heads. In retrieved specimens, we observed two distinct material loss patterns, described as Type 1 and Type 2. In Type 1 pattern tapers, as-manufactured regions of the taper can be observed on the distal and proximal portions of the taper with the material loss occurring between these regions. For Type 2 tapers, the as-manufactured regions of the taper are observed only on one end of the femoral head bore taper, typically the proximal region. In our validation study, the estimated volumetric material loss from the validation samples showed high correlation with gravimetric loss for Type 1 tapers (R^2^ > 0.995, slope = 1.015)(Appendix 1. Supplemental material is available with the online version of *CORR*^®^). Additionally, as part of this study, a sensitivity analysis showed that 24 profiles were sufficient to be within 1% of the gravimetric measurements for Type 1 tapers. The volume of material loss of the Type 2 heads also was estimated using the same described method for head bore taper measurement. Type 2 components have higher uncertainty in their volumetric material loss estimations compared with Type 1 because of fewer as-manufactured surfaces available for linear fitting (Appendix 1. Supplemental material is available with the online version of *CORR*^®^).

### Estimation of Material Loss from Stem Cone Tapers

To estimate the volume of material loss from the stem cone taper, the method used to estimate the material loss from head bore tapers was modified owing to the presence of “microgrooves.” In this study, stem cone tapers that had a surface topography with a periodic pattern, a wavelength greater than 100 μm, and an amplitude greater than 4 μm were considered “microgrooved,” as previously described [2]. Stem cone tapers that did not meet these criteria were considered “smooth” or “nonmicrogrooved.” For microgrooved surface topography, it is not possible for a least-squares straight line to represent the as-manufactured surface because the uncertainties introduced by this approximation may be larger than the volume of material loss. Furthermore, our experience has shown that some regions of microgrooves on stem cone tapers may have plastic deformation but no material loss or regions of iatrogenic damage, which need to be excluded from the estimation of material loss. Preliminary observations of stem cone tapers under optical microscopy also showed that in vivo material loss was seen in isolated regions (Appendix 2. Supplemental material is available with the online version of *CORR*^®^), unlike head bore tapers in which material loss may be seen in larger regions in contact with the stem. Owing to the difference observed in the patterns of material loss between head bore tapers and stem cone tapers, the method for estimation of material loss from head bore tapers was modified for stem cone tapers.

A Talyrond^®^ 585 roundness machine equipped with a diamond stylus was used to measure 360 equally spaced axial profiles on each stem cone taper to capture damage in each isolated region. Initially, the surface of the five stem cone tapers with the greatest damage was inspected using a scanning electron microscope (SEM) (SUPRA^®^ 50VP; Carl Zeiss NTS GmbH, Oberkochen, Germany) and optical microscopy (Keyence, Osaka, Japan) in conjunction with inspection of the measured profiles and surface maps (TalyMap, Taylor Hobson Ltd). This allowed for differentiation between fretting-corrosion damage and material loss, iatrogenic damage and material loss, and as-manufactured regions (Appendix 2. Supplemental material is available with the online version of *CORR*^®^). The measurement process, being a contact method with a diamond stylus, left microscopic scratches on the surface resulting from local plastic deformation. In some cases, the profiling process plowed through accumulated debris. After the appearance of these features under SEM had been correlated with the optical microscopy, subsequent inspections were done using optical microscopy and Talyrond^®^ profiles and surface maps, except when more detailed examination was required.

The stem cone taper microgrooves are axisymmetric with a small axial offset owing to the microgroove helix. For regions of tapers with identified fretting-corrosion material loss, the axial profiles were aligned with similar regions of profiles from the axial location without material loss. We tested the axisymmetry by aligning profiles measured in different locations around the circumference of the stem cone taper, which showed little or no damage (Appendix 2. Supplemental material is available with the online version of *CORR*^®^). The difference between the volume enclosed by the profiles projected over a 1° partial annulus with no material loss and profiles with fretting corrosion spanning equal radial slices was used to calculate total volumetric material loss from stem cone tapers. The area under the curve of each radial profile depends on the smooth or grooved topography of the stem cone. Equal depth less than 100 µm is used to capture changes in surface topography and material loss between profiles with damage and no damage. Material loss resulting from iatrogenic damage was excluded during estimation of volumetric material loss (Appendix 2. Supplemental material is available with the online version of *CORR*^®^).

### Taper Angle Clearance Estimation

The taper angle of each stem cone taper and head bore taper and thus the taper clearance, defined as the difference between the head bore taper angle and the stem cone taper angle, was estimated previously for each head-stem pair in this study [16]. Briefly, a stylus tip with a 2-mm diameter ruby sphere was used to measure five roundness profiles on the head bore tapers and stem cone tapers. The circumferential profiles were measured in the as-manufactured regions, if possible. Regions of asymmetric material loss or surface deposits were excluded from the analysis of each profile. The taper angle was estimated from the relative radius and relative height of the profiles.

### Statistical Analyses

Rates of volumetric material loss were examined using the Shapiro-Wilk test for normality and found to be nonparametric. Statistical analyses were performed using nonparametric methods using SPSS^®^ Statistics Version 23 (IBM, Armonk, NY, USA). Wilcoxon signed-rank test was used to calculate the significance between the rate of normalized volumetric material loss from the ceramic and CoCr cohorts. The Mann-Whitney U test was used to calculate the significance between the volumetric material loss from female and male tapers in the CoCr cohort. For correlations, we used the Spearman rank correlation test. Results were considered significant at a probability less than 0.05. In a previous study, a sample size of 100 was selected based on an a priori power analysis to detect a difference between the metal and ceramic cohorts in terms of visual fretting-corrosion score of 1 with 99% power [17]. Moreover, the current study was sufficiently powered (power = 80%; β = 0.2) to detect a moderate effect size (Spearman’s ρ = 0.25 or higher) with error probability α = 0.05 with the combined sample size of 100 (G*Power, Version 3.1.9.2; Heinrich-Heine-Universität Düsseldorf, Dusseldorf, Germany).

## Results

**Fig. 1:**
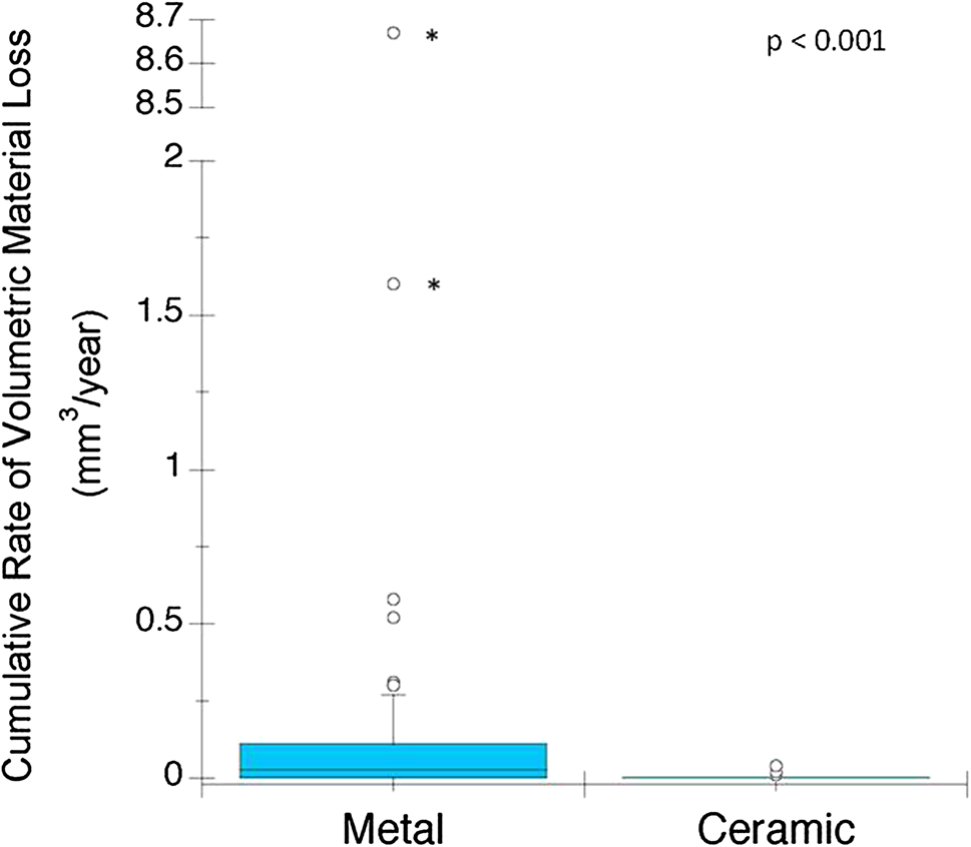
The box plot shows the rate of material loss from the metal and ceramic cohorts. The median and the maximum values seen for the CoCr cohort (median = 0.1 mm^3^, maximum = 9 mm^3^) are an order of magnitude greater compared with the ceramic cohort (median = 0.0 mm^3^, maximum = 0.4 mm^3^). Outliers with asterisks indicate a value taken from a Type 2 pattern of material loss.

**Fig. 2:**
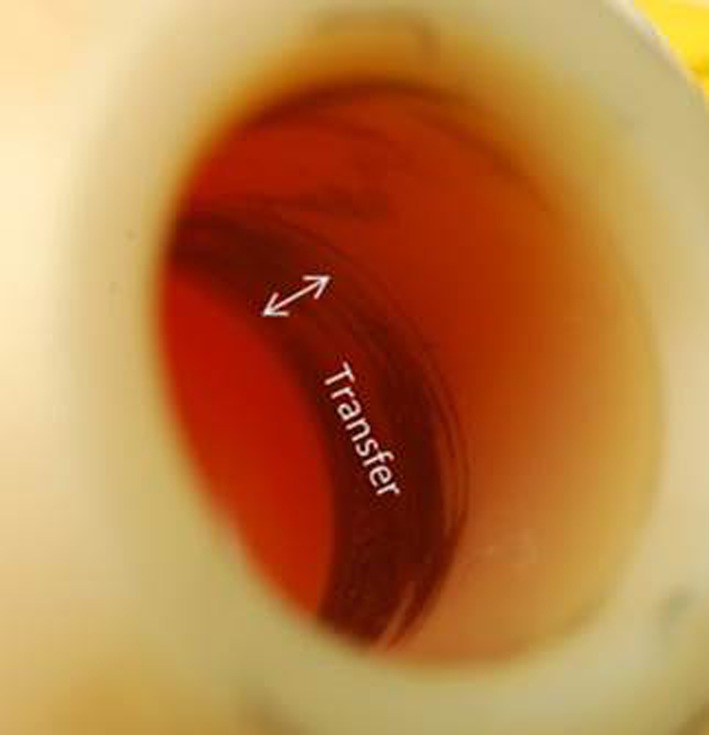
A region of metal transfer was observed on the proximal end of 42 of 50 of the ceramic tapers. For ceramic heads, the head bore taper and matching stem cone taper geometry are designed to have highest contact pressure at the proximal end.

In this study, the cumulative volumetric materials loss from ceramic taper junctions was significantly less than CoCr taper junctions (mean difference = 0.3 mm^3^; p < 0.001) (Fig. 1). Specifically, the cumulative volumetric material loss estimated for the ceramic cohort had a median of 0.0 mm^3^ per year (range, 0.0–0.4 mm^3^) and the CoCr cohort had a median of 0.1 mm^3^/year (range 0.0–9 mm^3^). This result was similar when Type 1 and Type 2 patterns were analyzed separately. For the CoCr cohort, 44 of 50 (88%) femoral heads had Type 1 pattern of material loss and the remaining six of 50 CoCr heads had a Type 2 pattern. Head-stem pairs with Type 1 pattern had median material loss 0.07 mm^3^ (range, 0.0–0.91 mm^3^/year). We did not observe evidence of fretting corrosion or material loss for the ceramic head bore tapers, but we did observe metallic material transfer or oxide corrosion debris on the head bore taper surface (Fig. 2). There was no detectable material gain in measured profiles of ceramic head bore tapers, even in those that had visual evidence of metal transfer.

**Fig. 3:**
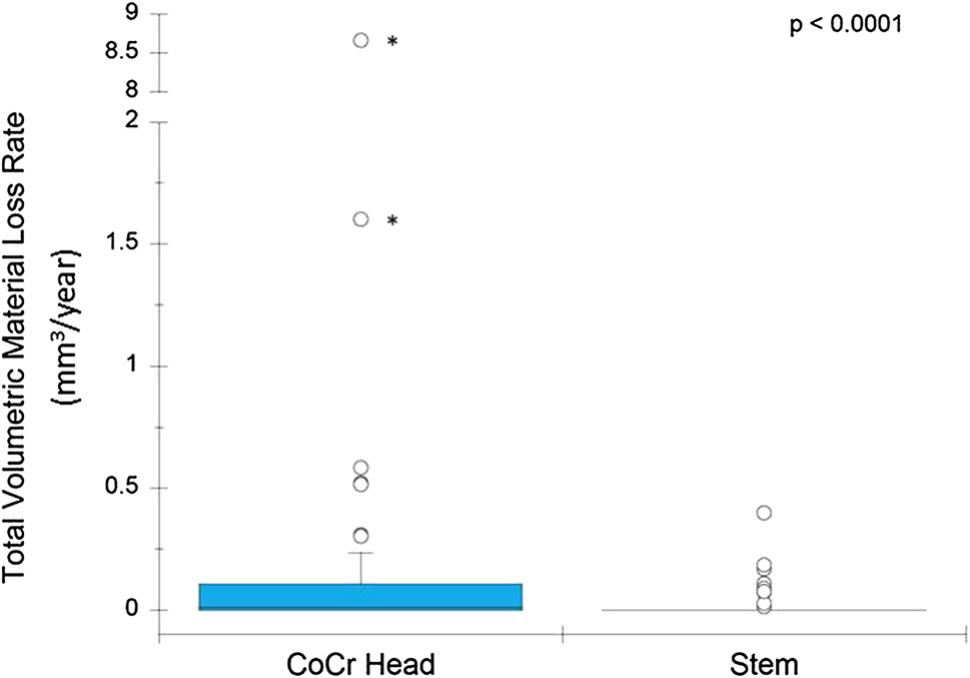
The box plot for rate of material loss at CoCr head bore and stem cone tapers shows a difference between head and stem surfaces. Outliers with asterisks indicate a value taken from a Type 2 pattern of material loss.

For the CoCr cohort, the majority of the cumulative material loss at the taper junction occurred on the head bore taper (Fig. 3) (p < 0.0001). Specifically, the femoral head bore tapers had a median material loss of 0.02 mm^3^ (range, 0.0–8.7 mm^3^/year), and the stem cone tapers had a median material loss of 0.0 mm^3^ (range 0.0–0.32 mm^3^/year). The majority of material loss in CoCr cohorts is from the femoral heads (more than 90%) as opposed to the stem tapers (p < 0.001) (Table 2). Moreover, the estimated volumetric material loss rate was greater in CoCr head bore tapers compared with the stem cone tapers (mean difference, 0.26 mm^3^). Inspection of the linear traces of the stem cone tapers revealed depths of material loss (range, 0–20 μm) similar to the head bore tapers (range, 0–35 μm, p = 0.19 [Mann-Whitney U test]). However, the fretting-corrosion damage was restricted to small, isolated areas on the stem cone tapers, resulting in less material loss. The outlier stem value in Table 2 with 2.5 mm^3^ of material loss had extensive intergranular corrosion and grain pullout. The depth of material loss for that stem from the Talyrond^®^ profiles was greater compared with other stems (> 100 µm). The implantation time for this component was 9 years and the rate of volumetric material loss was approximately 0.27 mm^3^ per year.

**Table 2:** Estimated total volumetric material loss and rate of volumetric material loss for both cohorts*

CoCr cohort Heads (n = 50)		CoCr cohort Stems (n = 50)		Ceramic cohort Heads (n = 50)		Ceramic cohort Stems (n = 50)	
Volume (mm^3^)	Rate (mm^3^/year)	Volume (mm^3^)	Rate (mm^3^/year)	Volume (mm^3^)	Rate (mm^3^/year)	Volume (mm^3^)	Rate (mm^3^/year)
0.04	0.02	0.00	0.00	0.00	0.00	0.00	0.00
0–4.34	0–8.67	0–2.5	0–0.32	0–0.03	0–0.04	0–0.74	0–0.37

* Data presented as median and range.

**Fig. 4:**
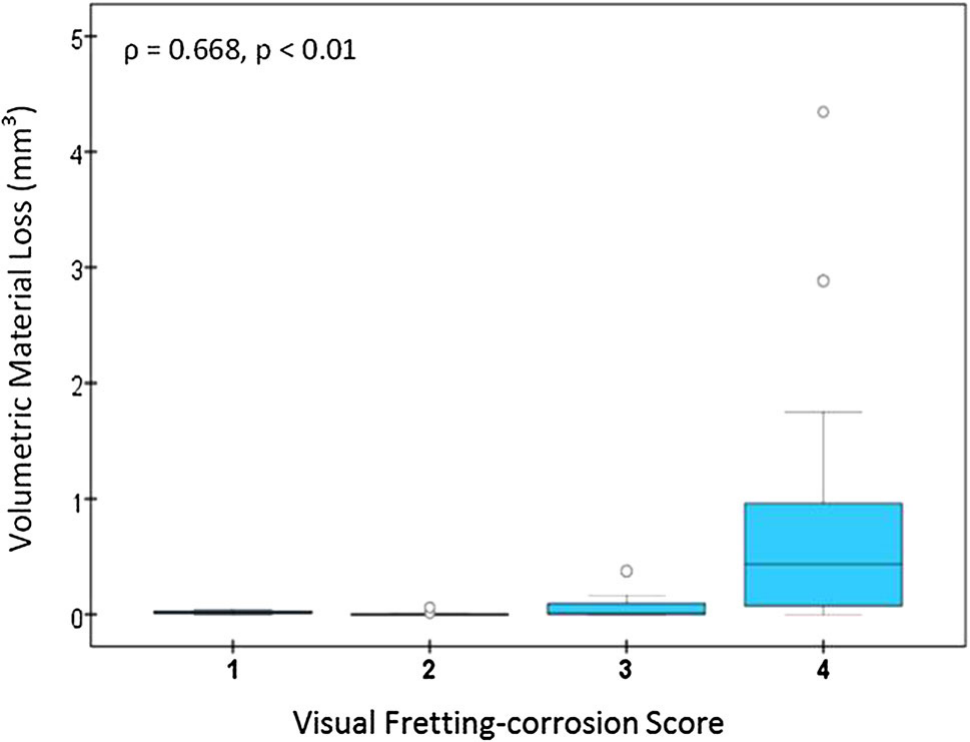
The correlation between visual fretting-corrosion score and estimated volumetric material loss in the CoCr cohort is shown.

There was a positive correlation between visual scoring and volumetric material loss (Spearman’s ρ = 0.668, p < 0.01) (Fig. 4). CoCr head bore tapers that had a score of 4 had the highest range of volumetric material loss (0–4.34 mm^3^, n = 21) followed by head bore tapers with a score of 3 (0–0.37 mm^3^, n = 12). CoCr head bore tapers scored 1 and 2 had the lower volumetric loss with ranges of 0 to 0.04 mm^3^ (n = 4) and 0 to 0.06 mm^3^ (n = 13), respectively.

With the numbers available, we did not observe any correlations between cumulative volumetric material loss and the available device factors including taper angle clearance (ρ = 0.06, p = 0.70), head size (ρ = 0.05, p = 0.72), head offset (ρ = 0.15, p = 0.29), lateral offset (ρ = 0.15, p = 0.29), stem taper material (Ti6Al4V, TMZF^®^, and CoCrMo alloys) (p = 0.71), and stem surface finish (p = 0.2). With the numbers available, we did not observe any correlations between the rate of cumulative material loss and patient factors including implantation time (ρ = 0.19, p = 0.18), patient age at implantation (ρ = −0.06, p = 0.35), activity levels (ρ = 0.15, p = 0.16), and BMI (ρ = 0.23, p = 0.07) (Table 3).

**Table 3:** Correlation between device and patient factors and cumulative rate of volumetric material loss in the CoCr cohort

Variable	Spearman’s correlation (ρ)	p value (significant if < 0.05)
Device factors		
Taper angle clearance* [16]	0.06	0.70
Absolute taper angle clearance* [16]	0.20	0.16
Head size	0.05	0.72
Head offset	0.15	0.29
Lateral offset	0.26	0.07
Stem taper material	–	0.71 (Kruskal-Wallis)
Stem taper surface finish	–	0.20 (Mann-Whitney U)
Patient factors		
Implantation time	0.19	0.18
Patient age at implantation	−0.06	0.35
BMI	0.23	0.07
UCLA Activity Score	0.15	0.16
Sex	–	0.06 (Mann-Whitney U)

* The absolute value of previously estimated taper angle clearance for head-stem junctions, looking at the effect of the net gap on material loss [16].

## Discussion

Fretting corrosion has been observed in retrieved femoral head-stem junctions since the introduction of modularity in hip arthroplasty; however, with the introduction of large-head MoM implants and implants with dual modularity there has been more interest in this phenomenon [4, 18, 19, 21, 24]. MoP bearings remain the historical gold standard in THA. Additionally, increased fracture resistance of latest generation ceramic bearings (CoP and CoC) has led to widespread adoption in the United States [20] and more than 50% in the United Kingdom and Australia [3, 22]. Fretting corrosion is still seen in retrieved head-stem tapers of modern MoP and ceramic bearings. There is no standardized method to measure volumetric material loss in tapers and no quantitative loss information available for designs other than large-head MoM. In this study, we estimated the volume of material loss from 100 paired explanted male stem cones and female head bore tapers subdivided into matched cohorts of 50 ceramic heads and 50 CoCr heads. This is the first study, to our knowledge, to quantify volumetric material loss from tapers other than large-head MoM designs. Total volumetric material loss in our CoCr cohort was an order of magnitude higher than the loss in the ceramic cohort. Femoral head material was the only factor that correlated with volumetric material loss, among the device and patient factors we investigated. These findings support the hypothesis that the use of ceramic heads mitigates metallic material loss from taper junctions. Visual fretting-corrosion scores were correlated with volumetric material loss

This study has several limitations. We used a method in this study that originally was developed to estimate material loss from Type 1 tapers and this method has greater uncertainty for the Type 2 tapers (Appendix 1. Supplemental material is available with the online version of *CORR*^®^). In the CoCr cohort, six of 50 (12%) head cone tapers are Type 2, with regions of an as-manufactured surface at only one end of the taper. For Type 2 tapers, the as-manufactured surface is estimated by extrapolating over the length of the taper from the as-manufactured region at one end of the taper, compared with the Type 1 taper in which the as-manufactured surface is estimated from interpolating between the two as-manufactured regions at each end of the taper. The extrapolation process, where the as-manufactured surface is estimated from the as-manufactured region at one end of the taper may lead to substantive uncertainties, particularly in cases where there is an unworn region of a few millimeters in length used to extrapolate over a taper that may be between 10 and 20 mm in length. Extrapolation from one end may lead to substantive uncertainty compared with having as-manufactured surfaces on either end. An uncertainty analysis for Type 2 tapers was beyond the scope of this study; however, measurements from the previously mentioned cohort of never-implanted femoral heads and taper adapter sleeves were reanalyzed as Type 2 tapers, excluding the available as-manufactured surfaces on the distal end. Estimation of material loss as Type 2 tapers, for the same samples, had a lower correlation with gravimetric measurements (Appendix 1. Supplemental material is available with the online version of *CORR*^®^).

Another limitation of this study, like all retrieval studies, is that it is a sample of clinical failures, and it does not necessarily reflect the performance for the population of well-functioning implants. The described validation method developed for head cone tapers in this study used new components. We selected ceramic implants for this study with the longest implantation time available in our retrieval collection at the time of selection; however, the study is limited to revised implants and the matching criteria [17]. Another limitation is a phenomenon seen in all surface profilometry studies using a diamond stylus. The contact measurement method induced submicron, visible scratches during measurement (Appendix 1. Supplemental material is available with the online version of *CORR*^®^). The surface is deformed by the same amount everywhere and was shown to be in the range of 20 to 40 nm, resulting in a true displaced resultant profile. The stylus tip displaced some debris attached to the surface during measurement. Debris is a mixture of oxide and biological products that has reattached to the surface after the reactions. Debris displacement from the surface did not affect the measurements of net material loss from the taper surfaces [23].

This matched cohort study found that the rate of material loss from head-stem tapers in MoP bearings is an order of magnitude higher compared with head-stem tapers in CoP and CoC bearings. To our knowledge, there are no previous studies examining the volumetric material loss from tapers including ceramic heads or MoP bearings, making comparisons with our study difficult. The material loss from the head bore tapers in our study is one order of magnitude lower compared with those reported in large-head MoM tapers. The magnitude of material loss is the same with the magnitude of material loss reported from liner backside (Table 4) [1, 4, 15, 18, 19]). Our study also showed that the majority of the material lost is from the head bore tapers and using ceramic femoral heads eliminates material loss from this surface (Table 4).

**Table 4:** Reported values of quantified material loss from head-stem tapers in previous studies

Measured surface	Bearing type	Study	Number of components	Mean volume (± SD)* (range) mm^3^	Mean rate (± SD)* (range) mm^3^/year
Female taper	CoCr heads (diameter < 40 mm)	Current Study	50	0.39 (± 0.83) (0–4.34)	0.29 (± 1.24) (0–8.67)
	Large-head MOM (diameter ≥ 40 mm)	Hothi et al. [15]	150	1.52 (0.13–25.89)	N/A
		Langton et al. [18]	111	N/A	Design I: 0.13 (0.01–3.15) Design II: 0.44 (0.02–8.34)
		Bishop et al. [4]	5	8.4 (2.6–20.2)	2.02 (0.6–4.9)
Male taper	CoCr heads (diameter < 40 mm)	Current Study	50	0.10 (± 0.37) (0–2.5)	0.04 (± 0.08) (0–0.32)
	Ceramic	Current Study	50	0.04 (±0.14) (0–0.74)	0.02 (± 0.08) (0–0.37)
	Large-head MOM (diameter ≥ 40 mm)	Matthies et al. [19]	36	0.29 (0–0.83)	0.08 (0–0.36)
		Bishop et al. [4]	2	0.03 (0.02–0.035)	0.01 (0.005–0.006)
Liner backside	Large-head MOM (diameter ≥ 40 mm)	Agne et al. [1]	21	0.4 (0–1.7)	0.2 (0–1.2)

* Added when available; SD available only for the current study; CoCr = cobalt chromium; MoM = metal on metal; N/A = not available.

To our knowledge, we investigated the largest number of stem cone tapers complete with mating femoral heads. We found that in the CoCr cohort, the stem cone tapers had one magnitude lower mean rate of material loss compared with head bore tapers. Previous studies also reported higher volumetric material loss from head bore tapers compared with stem cone tapers [4, 19]. Some researchers also have observed differences in patterns of material loss between components where stem cone tapers had damage in isolated regions unlike head bore tapers with bands of material loss around the taper [4, 18, 19]. These same researchers offered possible electrochemical and biomechanical explanations regarding why the pattern of material loss is prominently axisymmetric in head bore tapers and, if seen at all, is in localized areas on stem cone tapers; but the exact mechanism of the differences in the patterns of material loss between head bores and stem cones is unknown. In our study, the variability in the patterns of material loss and the different surface topographies (grooved or smooth) did not affect the sensitivity of measurement.

We found a positive correlation between the visual fretting-corrosion scores and the volumetric material loss for MoP bearings. A correlation between visual fretting-corrosion scores and volumetric material loss has been reported for large-head MoM bearings [15]; however, visual fretting-corrosion scoring is semiquantitative and does not provide a quantitative measure of the amount of material lost from the surface. Our visual fretting-corrosion scores were unable to differentiate in the high range of material loss in CoCr heads with moderate and severe visual fretting-corrosion scores (scores of 3 and 4) (Fig. 4). This finding is similar results seen in large-head MoM bearings [15]. In cases of severe fretting-corrosion damage, the severity of the discoloration seems to be unrelated to the actual material loss. Thus, although useful, visual fretting-corrosion scoring methods have limitations, and fully quantifying the amount of material loss at these interfaces may be more useful when analyzing fretting corrosion in the context of patient and device factors.

In our study, the only factor that we found that was associated with decreasing cumulative material loss from taper junctions was femoral head material. We found no correlation between cumulative rate of material loss from the taper junctions in the CoCr cohort and the stem material Ti6Al4V alloy, CoCr alloy, or TMZF^®^ alloy. There was no correlation between taper angle clearance and the volumetric material loss for the investigated cohorts. Taper angle clearance is positive or negative with proximal or distal engagement respectively [16]. To account for the effect of net clearance, we looked at the effect of absolute clearance on material loss, and found no correlation. To our knowledge, only one other study has investigated the effect of device factors and rate of volumetric material loss from large-head MoM bearings. Langton et al. [18] investigated two types of commercially available designs of large-head MoM bearings and found statistically significant (p < 0.05) correlations between rate of volumetric material loss and taper angle, head offset, distance (taper engagement level to center of rotation), and horizontal lever arm distance (lateral offset). Other studies which have quantified the volumetric material loss did not investigate the relationship between material loss and device design factors [15, 19]. One study which looked at the effect of device factors did not quantify the rate of material loss [11]. Moreover, with the numbers available, we did not observe any correlations between material loss and patient or device factors.

The use of ceramic heads with CoCrMo alloy stems appears to reduce the release of Co and Cr products from the taper junctions in this small matched-pair series. The use of a ceramic head with a titanium alloy stem should completely eliminate Co and Cr release. The results from our study show that ceramic head combinations decreased overall metal release caused by taper fretting and corrosion compared with MoP bearings. The majority of cumulative metal released from the taper junctions was from the CoCr femoral head bore taper. To our knowledge, this is the first study that quantifies material loss from taper junctions with MoP, CoC, and CoP bearings. Quantitative data provide comparable material loss information for future studies looking at different device and material factors. It also might be useful for correlations between systemic cytotoxicity with volumetric material loss. Provided a titanium alloy stem is used, the corrosion products are considered to be less cytotoxic than Co and Cr [8, 13]; however, more information is needed to determine the long-term clinical effects. The reduction of corrosion products makes ceramics a potentially attractive bearing for adverse local tissue reaction revisions [6, 7]. The most recent annual report from the Australian Orthopaedic Association National Joint Replacement Registry showed that the risk of ceramic fracture using new-generation ceramic composite heads is extremely low, 0.17 per 10,000 (0.0017%) [3]. Overall, the decision regarding which bearing combination to use in clinical practice for primary and revision THAs is complex and based on a host of factors including risk of fracture, bearing noise, cost, polyethylene wear, and metal alloy corrosion concerns. Our study contributes to the decision process by providing evidence that ceramic heads do not appear to have the same level of stem or bore fretting-corrosion concerns as with metallic heads.

## Electronic supplementary material

Below is the link to the electronic supplementary material.

Supplementary material 1 (DOC 3582 kb)

Supplementary material 2 (DOC 3060 kb)
