# Supplementary material for: Ceramic Heads Decrease Metal Release Caused by Head-taper Fretting and Corrosion
Source: Clin Orthop Relat Res. 2016 Feb 4;474(4):985–94. doi: 10.1007/s11999-015-4683-1 (PMC4773353; doi:10.1007/s11999-015-4683-1)
Supplement: Supplementary file 1 — Supplementary material 1 (DOC 3582 kb) [file 11999_2015_4683_MOESM1_ESM.doc]

| **Appendix 1**. Method to quantify volumetric material loss from retrieval female taper surfaces with fretting-corrosion damage using in vitro simulated surfaces for method validation. | | |
| --- | --- | --- |
| Step | Method | Figures |
| Visual fretting-corrosion scoring | Visual scoring method developed by Goldberg et al. or modified methods are used in the literature to evaluate fretting-corrosion severity on taper surfaces using a scale of 1 to 4, with 1 being the least severe and 4 being the most severe. | 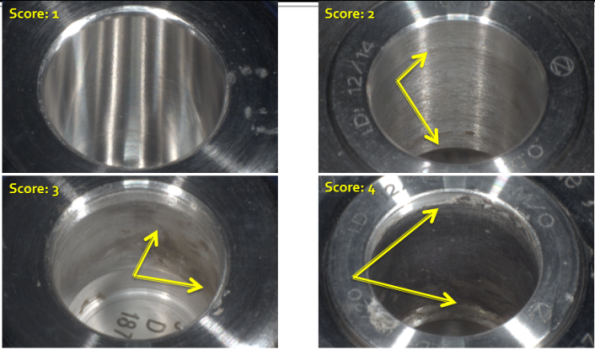 |
| Identifying patterns of material loss | Surface profile measurements (red dotted lines) of retrieval taper surfaces are a quantitative representation of surface damage. They also show that as-manufactured surfaces are present in retrievals which can be used to estimate the entire as-manufactured surface through mathematical fitting. | 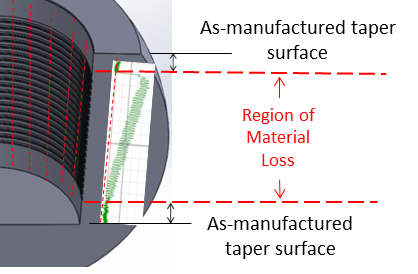 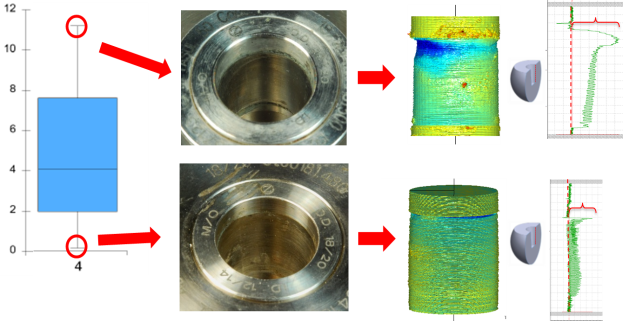 **Fretting-corrosion Score = 4**  **Estimated Wear Volume**  **[mm^3^]**  **55 µm**  **6 µm** |
| Comparing visual scores and surface profiles | Retrievals with a visual score of 4 have wide range of volumetric loss and depth of material loss in their surface profiles. Visual scoring is semiqualitative and has limitations in estimating the severity of fretting-corrosion damage. Surface profiles are quantitative representations of fretting-corrosion damage and material loss. |  |
| Method development – simulating material loss in vitro | A quantitative method to estimate the severity of material loss in vitro was developed using surface profiles. Never implanted taper adapter sleeves and CoCr femoral heads were machined to simulate the same patterns and depth of material loss seen in retrievals. | 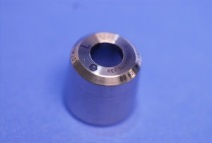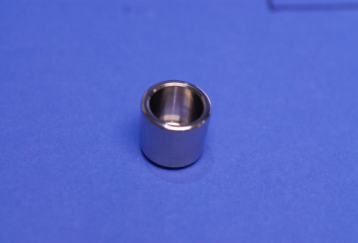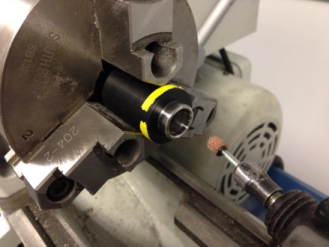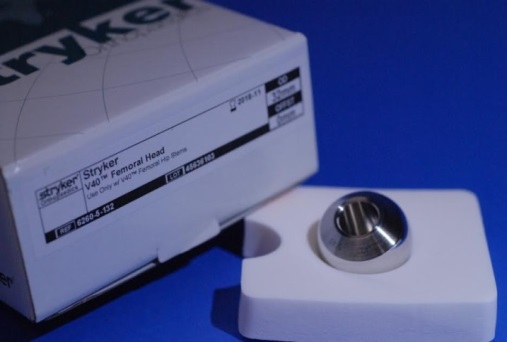 |
| Measuring simulated surfaces | Taper surfaces with simulated material loss were measured using a roundness machine (Talyrond® 585, Taylor Hobson Ltd, Leicester, UK) equipped with a diamond stylus. 24 equally spaced axial profiles (red dotted lines) were measured around each taper surface. | 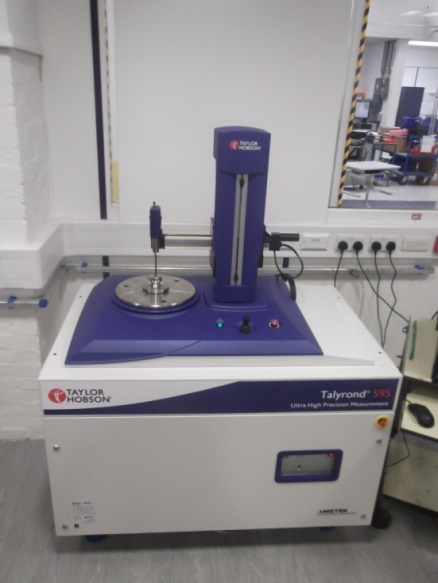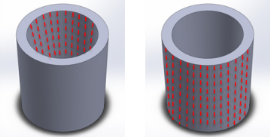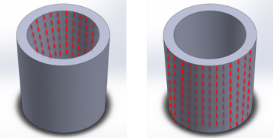 |
| Identifying regions of as-manufactured surfaces | A trained user identified regions of “as-manufactured” surface on each profile. | 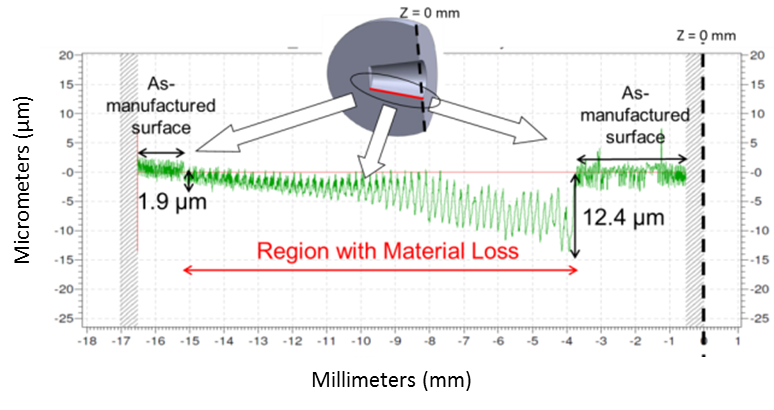 |
| Estimating as-manufactured surfaces with LS linear fitting | Measured profiles were imported into MATLAB® (MathWorks®, Natick, MA, USA). A custom script was used with the in-built polyfit function for least-squares (LS) linear fitting. Only user specified regions of as-manufactured surfaces were used for fitting. The fitted line is the reference, estimated as-manufactured surface for volumetric material loss calculation. | 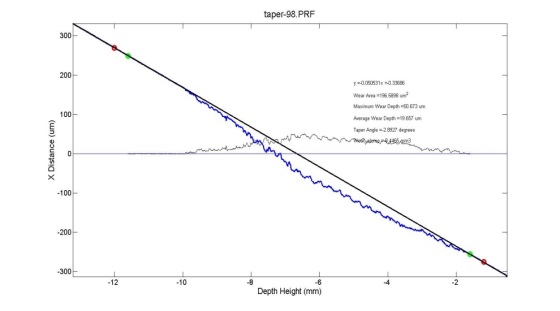 Region with Material Loss  As-manufactured surfaces 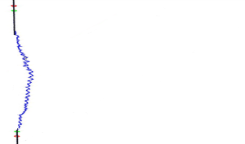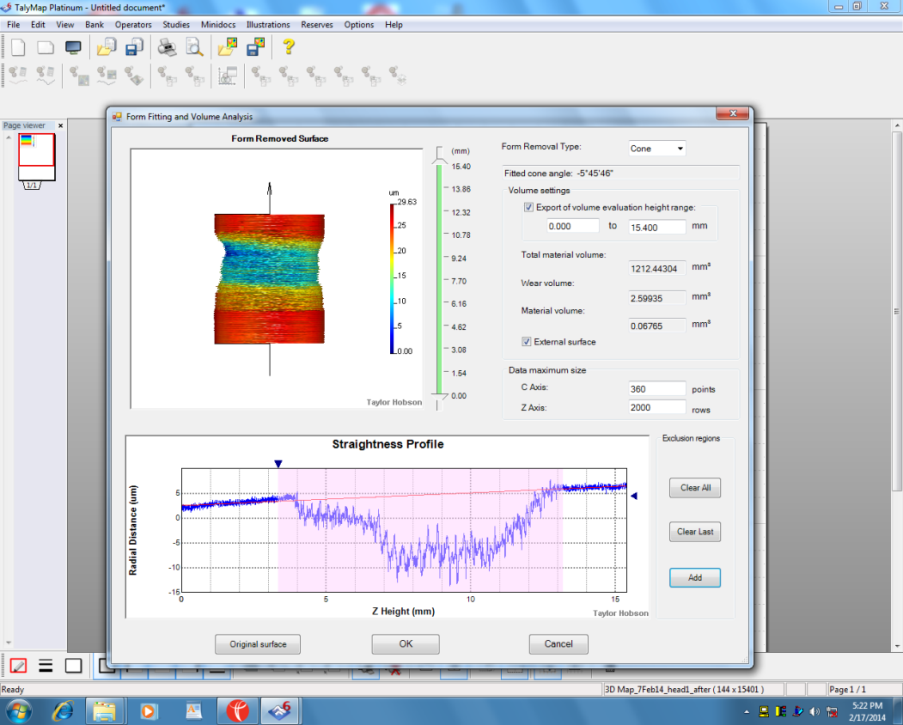 |
| Estimating volumetric material loss | The LS fit was established as the estimated as-manufactured surface (1). Integral rectangular areas of material loss were calculated using the spacing between each data point and the distance between the measured surface and the estimated as-manufactured surface (2). Each rectangle is used to calculate the volume of a partial annulus based on the taper local radius, known from axis alignment before profile measurements (3). All partial annuli were summed to estimate the volume of material loss in the taper (4). | 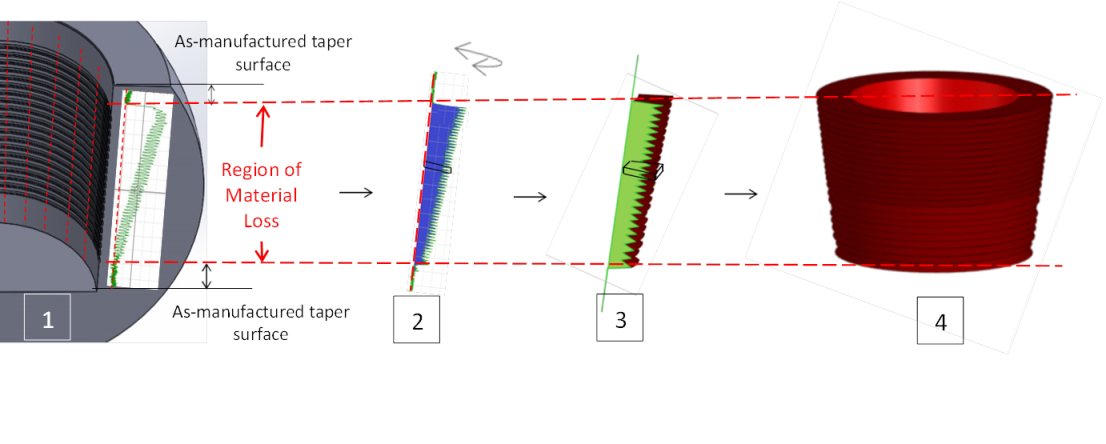  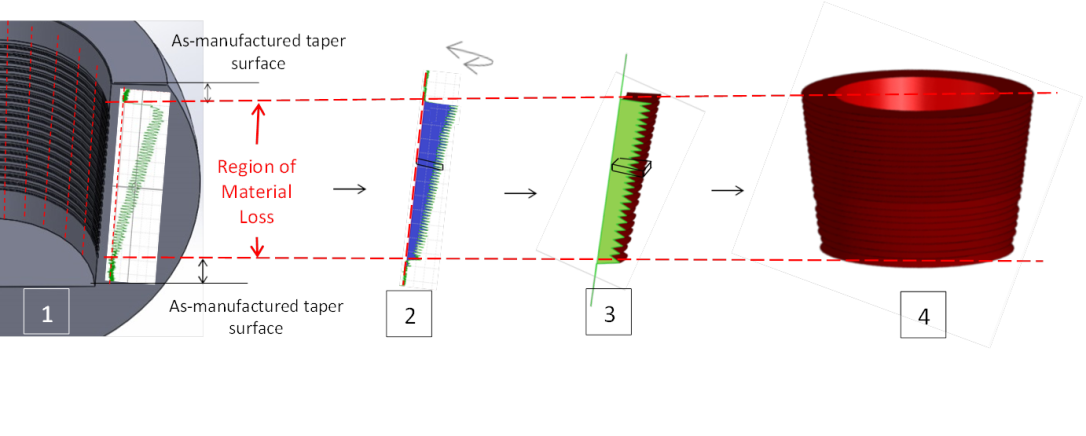 |
| Validating method | There was a high correlation between estimated volumetric material loss calculated using the MATLAB® script and the gravimetric measurements of volumetric material removal (R^2^ > 99.5%, Slope = 1.0149). | 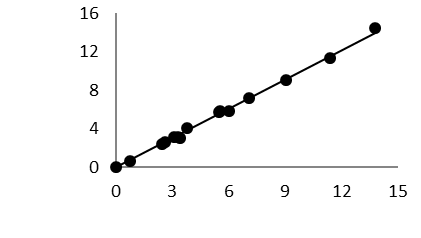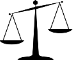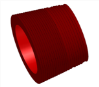 **y = 1.0149x**  **R^2^ = 0.9965** |
| Comparing Type 1 and Type 2 patterns | Volumetric material loss values calculated for Type 2 heads had higher uncertainty using this method because as-manufactured surfaces were available on only one side of taper for Type 2. The correlation between gravimetric measurements and Type 2 analyses is lower compared with Type 1  (R^2^ = 94.3%, Slope = 0.94). | 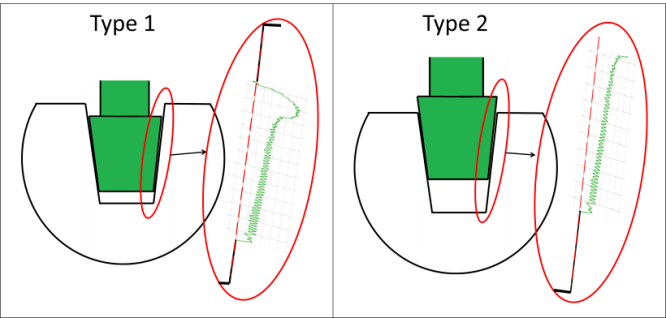 |

1. Goldberg JR, Gilbert JL, Jacobs JJ, Bauer TW, Paprosky W, Leurgans S. A multicenter retrieval study of the taper interfaces of modular hip prostheses. *Clin Orthop Relat Res.* 2002;401:149-161.

2. Higgs GB, Hanzlik JA, MacDonald DW, Gilbert JL, Rimnac CM, Kurtz SM, Implant Research Center Writing Committee. Is increased modularity associated with increased fretting and corrosion damage in metal-on-metal total hip arthroplasty devices?: a retrieval study. *J Arthroplasty.* 2013;28(8 suppl):2-6.
