# Supplementary material for: Ceramic Heads Decrease Metal Release Caused by Head-taper Fretting and Corrosion
Source: Clin Orthop Relat Res. 2016 Feb 4;474(4):985–94. doi: 10.1007/s11999-015-4683-1 (PMC4773353; doi:10.1007/s11999-015-4683-1)
Supplement: Supplementary file 2 — Supplementary material 2 (DOC 3060 kb) [file 11999_2015_4683_MOESM2_ESM.doc]

| 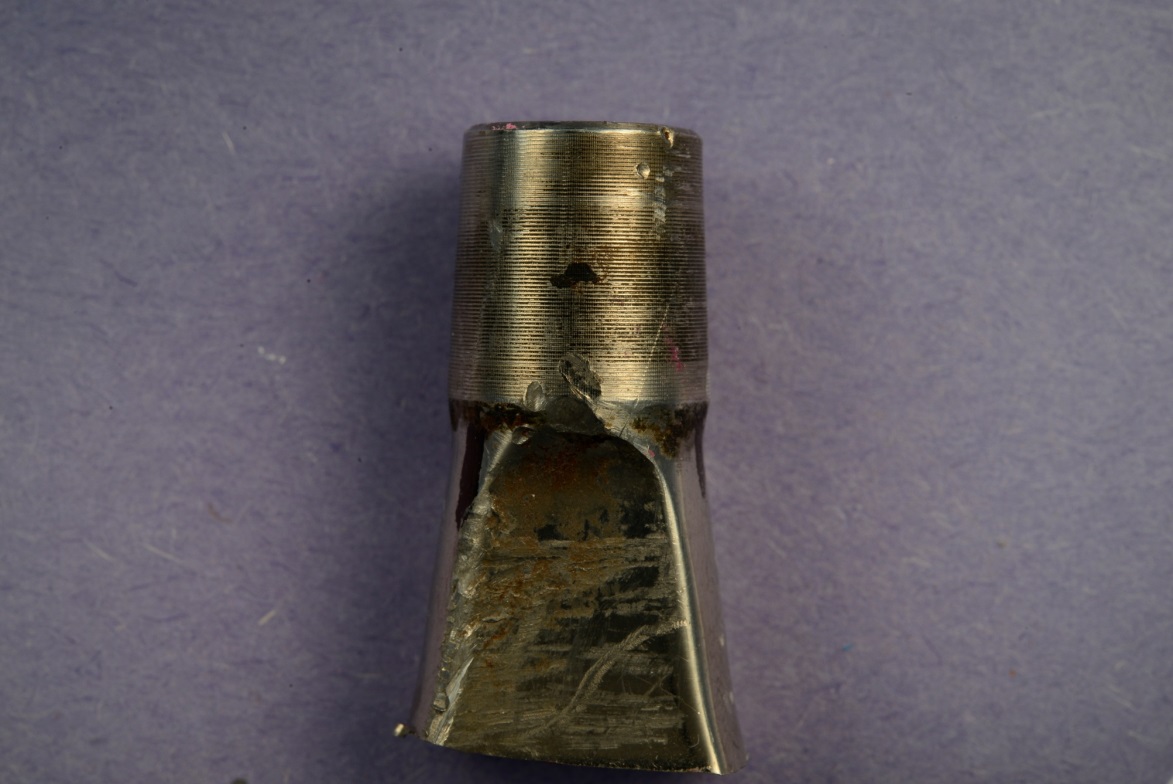**Appendix 2**. Method to quantify volumetric material loss from retrieval male taper surfaces with fretting-corrosion damage. | | |
| --- | --- | --- |
| Step | Method | Figures |
| Identifying regions with iatrogenic damage | Stem cone tapers have risk of getting iatrogenic damage during removal surgery (regions in white boxes). Iatrogenic damage needs to be identified and eliminated from material loss calculation. |  |
| Measuring retrieval tapers | Tapers were measured using a roundness machine (Talyrond® 585, Taylor Hobson Lrd, Leicester, UK) equipped with a diamond stylus. 360 equally spaced axial profiles (red dotted lines) were measured around each taper surface to form a surface map and capture isolated regions of in vivo material loss. | 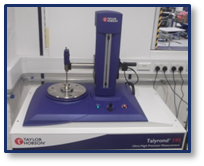 |
| Matching observations with optical microscopy with regions on measured profiles | Surface mapping using 360 profiles detected the changes in morphologic features of the surface in isolated regions for material loss estimation. Each profile is inspected for regions of material loss. Optical microscopy is used to eliminate regions that have iatrogenic damage from the material loss analysis. | 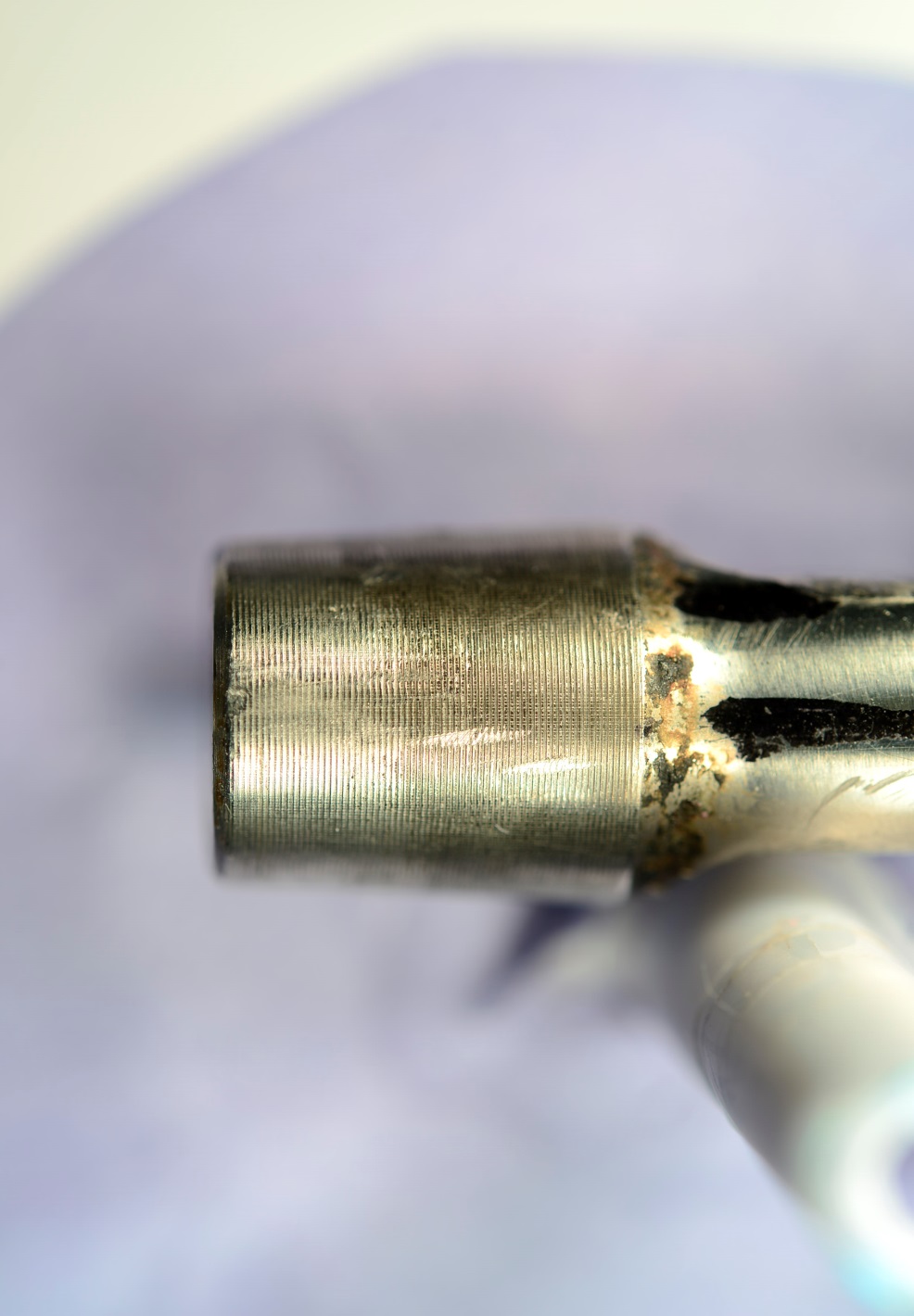 No damage  In vivo  Iatrogenic |
| SEM imaging identified regions with damage | SEM imaging was used to cross-check the mode of damage identified using optical microscopy and measured profiles. The location and appearances of features for in vivo and iatrogenic damage seen in the SEM were matched with features seen in the optical microscope, the latter which was used for subsequent inspections. | No damage  In vivo  Iatrogenic  1  2  3 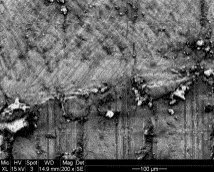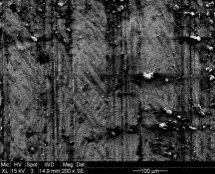 1  2 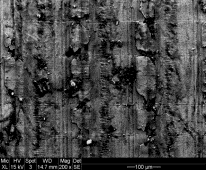 3  Iatrogenic Damage  Flattened Microgroove  Surfaces  Machining Marks  100µm  100µm  100µm 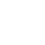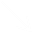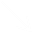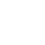 Machining Marks 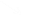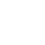 |
| Identify regions without material loss | Profiles without material loss on the same stem taper, or regions without loss, is the most accurate way to calculate volumetric material loss on male tapers. The circumferential overlay allows for alignment of peaks and valleys for identification of regions with material loss. | 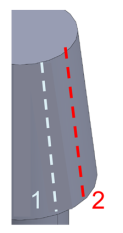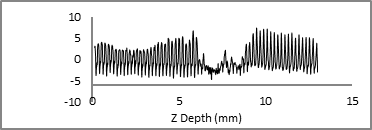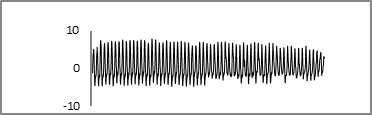 2  Profile with material loss  Profile without material loss  1  Surface Height (µm) 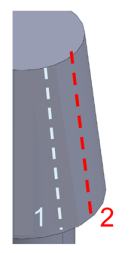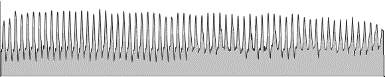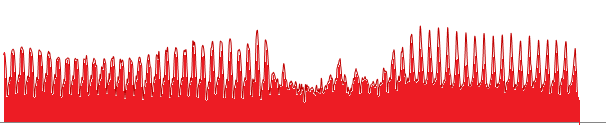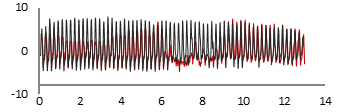 Surface Height (µm) |
| Calculation of area of material loss | The area under the curve of each radial profile depends on the smooth or grooved topography of the stem cone. Equal depth of surface height < 100 µm is used to capture changes in surface topography and material loss between profiles with damage and no damage. Material loss resulting from iatrogenic damage was excluded during estimation of volumetric material loss. | Z Depth (mm)  1  2 |
| Calculation of volumetric material loss | The difference between the volume enclosed by the profiles projected over a 1° partial annulus with no material loss and profiles with fretting-corrosion spanning equal radial slices was used to calculate total volumetric material loss from stem cone tapers. This difference is multiplied by the number of degrees (d) the type of deformation is observed in profiles and optical microscopy. | 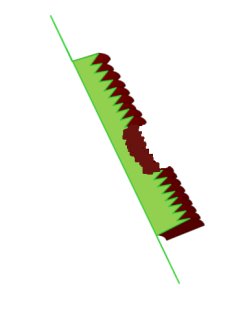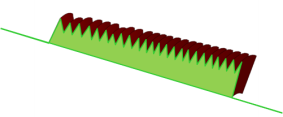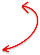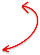 d°  d°  d° 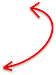 |
